# Effect of dietary palm olein oil on oxidative stress associated with ischemic-reperfusion injury in isolated rat heart

**DOI:** 10.1186/1471-2210-4-29

**Published:** 2004-11-09

**Authors:** Deepak Narang, Subeena Sood, Mathew Kadali Thomas, Amit Kumar Dinda, Subir Kumar Maulik

**Affiliations:** 1 Department of Pharmacology, All India Institute of Medical Sciences, New Delhi-110029, India; 2 Department of Pathology, All India Institute of Medical Sciences, New Delhi-110029, India

## Abstract

**Background:**

Palm olein oil (PO), obtained from refining of palm oil is rich in monounsaturated fatty acid and antioxidant vitamins and is widely used as oil in diet in many parts of the world including India. Palm oil has been reported to have beneficial effects in oxidative stress associated with hypertension and arterial thrombosis. Oxidative stress plays a major role in the etiopathology of myocardial ischemic-reperfusion injury (IRI) which is a common sequel of ischemic heart disease. Antioxidants have potent therapeutic effects on both ischemic heart disease and ischemic-reperfusion injury. Information on the effect of PO on ischemic-reperfusion injury is, however, lacking. In the present study, the effect of dietary palm olein oil on oxidative stress associated with IRI was investigated in an isolated rat heart model. Wistar rats (150–200 gm) of either sex were divided into three different groups (n = 16). Rats were fed with palm olein oil supplemented commercial rat diet, in two different doses [5% v / w (PO 5) and 10% v / w (PO 10) of diet] for 30 days. Control rats (C) were fed with normal diet. After 30 days, half the rats from each group were subjected to *in vitro *myocardial IRI (20 min of global ischemia, followed by 40 min of reperfusion). Hearts from all the groups were then processed for biochemical and histopathological studies. One way ANOVA followed by Bonferroni test was applied to test for significance and values are expressed as mean ± SE (p < 0.05).

**Results:**

There was a significant increase in myocardial catalase (CAT), superoxide dismutase (SOD) and glutathione peroxidase (GPx) activities with no significant change in myocardial thiobarbituric acid reactive substances (TBARS) only in group PO 5 as compared to group C. There was no light microscopic evidence of tissue injury. A significant rise in myocardial TBARS and depletion of myocardial endogenous antioxidants (SOD, CAT and GPx) along with significant myocyte injury was observed in control rats subjected to ischemia-reperfusion (C IR). Hearts from palm olein oil fed rats subjected to ischemia-reperfusion (PO 5 IR and PO 10 IR) were protected from increase in TBARS and depletion of endogenous antioxidants as compared to C IR group. No significant myocyte injury was present in the treated groups.

**Conclusions:**

The present study demonstrated for the first time that dietary palm olein oil protected rat heart from oxidative stress associated with ischemic-reperfusion injury.

## Background

Ischemic heart disease (IHD) is a major cause of death all over the world. Reduction in the blood flow to myocardium leads to IHD and its restitution (reperfusion), spontaneously or by drug / surgery, is essential for tissue/organ survival. However, reperfusion itself exacerbates myocardial injury, commonly known as myocardial ischemic-reperfusion injury (IRI) [1]. Therefore, IRI is considered as a common sequel of IHD. Oxidative stress has been largely implicated in the etiopathogenesis of IRI. Oxidative stress occurs due to increased production of reactive oxygen species (ROS) like, superoxide radical, hydrogen peroxide, hydroxyl radical at the time of reperfusion, which overwhelms the endogenous antioxidant defense [2]. Interaction of ROS with cell membrane and various other cellular components have deleterious effects on cellular functions and viability. Oxidative stress is evidenced by increased cellular accumulation of lipid peroxides and depletion of endogenous antioxidants [3-5].

Living organisms have developed antioxidant defense mechanisms against damage due to oxidative stress. These mechanisms in the heart have been extensively studied and the most active endogenous antioxidants involved in this process are superoxide dismutase (SOD), catalase (CAT), and glutathione peroxidase (GPx) [2,6]. In addition to this, alpha-tocopherols or vit E, vitamin C and beta-carotene constitute important exogenous antioxidants present in diet [7,8].

The physiological actions of diet continue to be the focus of interest because of its major role in ischemic heart disease. Dietary antioxidants e.g., vitamin E, beta-carotene, vitamin C have beneficial effects in oxidative stress associated with various cardiovascular diseases, including ischemic heart disease [9-11]. Therefore, dietary antioxidants have potential therapeutic role in the prevention and treatment of ischemic heart disease.

Palm oil, obtained from the fruit of the tropical plant *Elaeis guineensis*, is the second major edible oil used worldwide [12]. Palm olein oil (PO), a liquid fraction obtained from the refining of palm oil, is rich in oleic acid (42.7–43.9%), beta-carotene and vitamin E (tocopherols and tocotrienols). PO is used as dietary oil in many parts of the world including India. In some previous studies, palm oil has been reported to have antioxidant effects in hypertension [13,14] and arterial thrombosis [15] in rats. In addition to this, palm oil has been shown to increase prostacyclin (PGI_2_) and reduce thromboxane A_2 _(TXA_2_) levels in tissues [16]. However scientific studies on antioxidant effects of palm olein oil on ischemic heart disease and ischemic-reperfusion injury are still lacking.

Therefore, the present study was designed to evaluate the effects of dietary palm olein oil on myocardial endogenous antioxidants and on oxidative stress associated with ischemic-reperfusion injury in isolated rat heart model.

## Results

There was no mortality, changes in body weight as well as food and water intake pattern of rats in any group.

### Biochemical parameters

#### I. Changes in the basal level of myocardial lipid peroxidation and endogenous antioxidants (Table 1)

**Table 1:** Effect of dietary palm olein oil on myocardial TBARS, catalase, SOD, and GPx levels in different groups

**Group**	**TBARS (nmol/mg protein)**	**CATALASE (U/mg protein**	**SOD (U/mg protein**	**GPx (U/mg protein)**
Control	7.8 ± 0.4	34.4 ± 2.1	3.5 ± 0.08	0.13 ± 0.01
Control IR	9.4 ± 0.3*	29.1 ± 0.8*	3.01 ± 0.15*	0.11 ± 0.002**
PO 5	8.5 ± 0.8	50.2 ± 3.5**	5.6 ± 0.5**	0.18 ± 0.01**
PO 10	8.2 ± 1.2	45.6 ± 4.0*	3.8 ± 0.3	0.16 ± 0.02
PO 5 IR	6.8 ± 0.4^+++^	56.9 ± 4.4^+++^	3.1 ± 0.2	0.12 ± 0.01
PO 10 IR	5.9 ± 0.6^+++^	36.3 ± 5.6	3.4 ± 0.3	0.15 ± 0.01^++^

All values are expressed as Mean ± SE (n = 6)

p values: * < 0.05; ** < 0.01; *vs *Control; ^++ ^< 0.01; ^+++ ^< 0.001 *vs *Control IR (one way ANOVA)

##### I.a. Basal myocardial TBARS levels

There were no significant changes in myocardial TBARS levels in both PO5 (8.5 ± 0.8 nmol / mg protein) and PO10 (8.2 ± 1.2 nmol / mg protein) groups when compared to that of control group (7.8 ± 0.4 nmol / mg protein).

##### I.b. Basal myocardial catalase (CAT) activity

There was a significant increase in myocardial CAT activity in both PO5 (50.2 ± 3.5 units / mg protein; p < 0.01) and PO10 (45.6 ± 4.0 units / mg protein; p < 0.05) groups as compared to that of control group (34.4 ± 2.1 units / mg protein).

##### I.c. Basal myocardial superoxide dismutase (SOD) activity

There were a significant increase in myocardial SOD activity in PO 5 (5.6 ± 0.5 units / mg protein; p < 0.01) group when compared to that of the control group (3.5 ± 0.08 units / mg protein). There was no significant change in myocardial SOD activity in PO10 (3.8 ± 0.3 units / mg protein) group.

##### I.d. Basal myocardial glutathione peroxidase (GPx) activity

There was a significant (p < 0.01) increase in myocardial GPx activity in PO5 (0.18 ± 0.01 units / mg protein) group when compared to that of the control group (0.13 ± 0.01 units / mg protein). There was no significant change in myocardial GPx activity in PO10 (0.16 ± 0.02 units / mg protein) group.

#### II. Changes in myocardial lipid peroxidation and endogenous antioxidants following ischemic-reperfusion injury (Table 1)

##### II.a. Myocardial TBARS levels after ischemic-reperfusion injury

There was a significant (p < 0.05) increase in myocardial TBARS level in the C IR group (9.4 ± 0.3 nmol / mg protein) when compared to that of the control group (7.8 ± 0.4 nmol / mg protein). There was a significant (p < 0.01) decrease in myocardial TBARS levels in both PO5 IR (6.8 ± 0.4 nmol / mg protein) and PO10 IR (5.9 ± 0.6 nmol / mg protein) groups when compared to that of the C IR group.

##### II.b. Myocardial CAT activity after ischemic-reperfusion injury

There was a significant (p < 0.05) decrease in myocardial CAT activity (29.1 ± 0.8 units / mg protein) in C IR group when compared to that of the control group (34.4 ± 2.1 units / mg protein). There was a significant rise in CAT activity in PO5 IR (56.9 ± 4.4 units / mg protein; p < 0.001) group with no significant change in PO10 IR (36.3 ± 5.6 units / mg protein) group when compared to C IR group.

##### II.c. Myocardial SOD activity after ischemic-reperfusion injury

There was a significant decrease in myocardial SOD activity in C IR group (3.01 ± 0.15 units / mg protein; p < 0.05) as compared to control group (3.5 ± 0.08 units / mg protein). No significant changes in SOD activities were observed in both PO5 IR (3.1 ± 0.2 units / mg protein) and PO10 IR (3.4 ± 0.3 units / mg protein) groups as compared to C IR group.

##### II.d. Myocardial GPx activity after ischemic-reperfusion injury

There was a significant (p < 0.01) decrease in myocardial GPx activity in C IR group (0.11 ± 0.002 units / mg protein) as compared to control group (0.13 ± 0.004 units / mg protein). There was no significant change in myocardial GPx activity in PO5 IR group (0.12 ± 0.01 units / mg protein) with a significant (p < 0.01) increase in myocardial GPx activity in PO10 IR group (0.15 ± 0.01 units / mg protein) as compared to C IR group.

### Histopathological study

**Figure 1:**
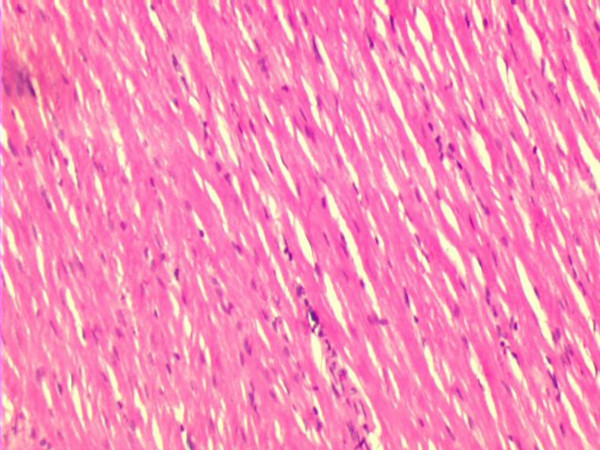
Light micrograph of heart tissue. Control rat heart (C) showing normal architecture (H & E X 10).

**Figure 2:**
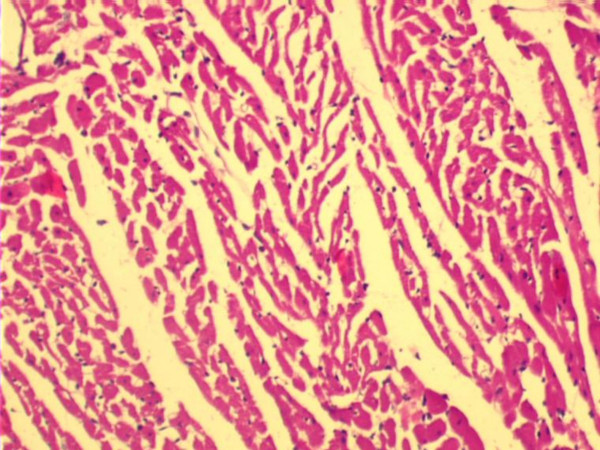
Light micrograph of heart tissue. Control rat heart subjected to 20 min ischemia and 40 min reperfusion (C IR) showing marked edema and focal destruction of myocardial fibres (H & E X 10).

**Figure 3:**
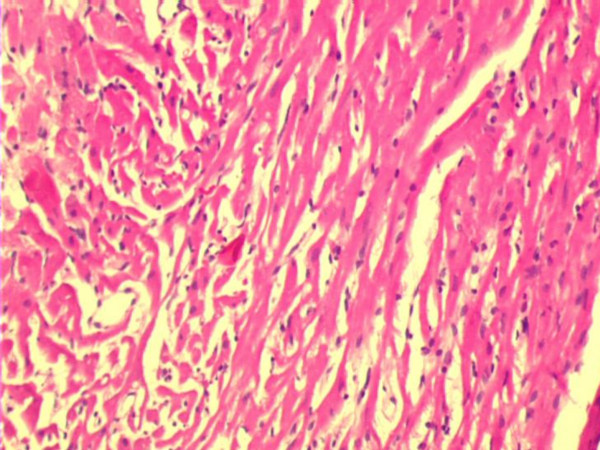
Light micrograph of heart tissue. Rat heart supplemented with 5% v/w of dietary palm olein oil subjected to 20 min ischemia and 40 min of reperfusion (PO5 IR) showing mild edema with occasional loss of myofibre (H & E X 10).

**Figure 4:**
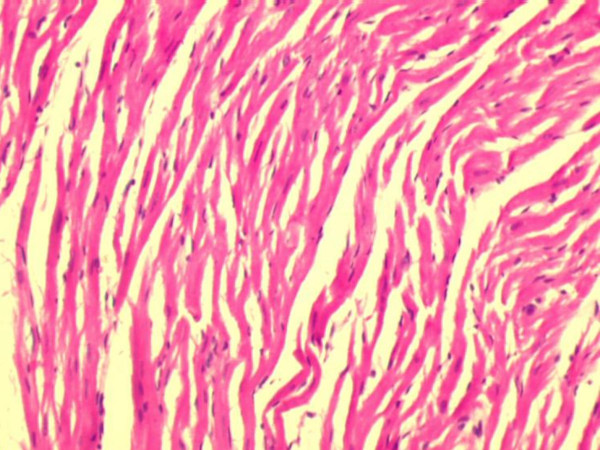
Light micrograph of heart tissue. Rat heart supplemented with 10% v/w of dietary palm olein oil subjected to 20 min ischemia and 40 min of reperfusion (PO10 IR) with mild to moderate edema and occasional loss of myofibre (H & E X 10).

Fig. 1 shows the H&E micrograph of control heart with normal architecture. In PO5 and PO10 groups there was no evidence of cellular injury (not shown). Focal loss of myocardial fibres and marked edema was observed in C IR group (Fig. 2). Mild to moderate edema was observed in PO10 IR group (Fig. 4). Degree of edema was reduced in PO5 IR with no evidence of focal necrosis (Fig. 3)

## Discussion

In the present study, a significant increase in myocardial SOD, catalase and GPx activity was observed in the lower dose of palm olein oil fed rats. However, their further augmentation was not observed in the higher dose, i.e., a dose dependent effect was not observed. The finding correlates with the previous studies in which an increase in response was not observed with the increase in the dose of supplemented vitamin E [26,27]. The possible reasons behind the lack of dose response relationship may be a decrease in intestinal absorption as a result of increase in dose [28] and newly absorbed vitamin E in part replacing the alpha-tocopherol in circulating lipoproteins [29].

Augmentation of endogenous antioxidants (SOD, CAT, GPx) has been recognized as an important pharmacological property, present in natural as well as many synthetic compounds [30-33]. This constitutes a major mechanism of protection against oxidative stress, offered by them [30,35,37]. The most abundant reactive oxygen species generated in living system is superoxide radical which is acted upon by SOD to produce hydrogen peroxide which in turn is inactivated by catalase and / or GPx into water and oxygen. Thus an increase in both SOD and catalase along with GPx activity is considered to be more beneficial in the event of oxidative stress [34].

Increase in myocardial TBARS and depletion of myocardial endogenous antioxidants support the occurrence of oxidative stress in the control hearts following ischemia-reperfusion in the present study. It was also accompanied by tissue injury with marked edema and focal loss of myocardial fibres. Similar changes have been reported earlier to occur following brief period of ischemia followed by reperfusion in rat heart [35-37]. Hearts from palm olein oil fed rats in both doses were protected against oxidative stress, as evidenced by inhibition of increase in TBARS, depletion of catalase, GPx and tissue injury following ischemia-reperfusion. In a previous study, palm oil has been reported to prevent oxidative stress induced hypertension in rats [13]. The mechanism of such protection can be attributed to the augmented endogenous antioxidant reserve of heart in the lower dose. However, the higher dose, which did not cause any significant augmentation of endogenous antioxidants, also inhibited depletion of antioxidants, rise in TBARS and tissue injury. It is possible that direct antioxidant effects of palm olein oil may be attributable to the presence of alpha tocopherols and tocotrienols, which are known to protect against oxidative stress.

Experimental as well as clinical studies with exogenous antioxidants supplementation have been shown to have protective effect in ischemic heart disease [38,39]. In this regard, the most commonly used exogenous antioxidants are vitamin E (tocopherols and tocotrienols), beta-carotene and vitamin C. Palm oil is also beneficial in conditions like hypertension [13,14,40], arterial thrombosis [15,41] and causes increase in PGI_2 _/TXA_2 _ratio [16]. Palm oil derived vitamin E rich in tocotrienols has shown beneficial effects against hypercholesterolemia [42,43] and is considered to be more potent than tocopherols [44].

The observations made in the present study have important nutritional significance for palm olein oil in relation to ischemic heart disease. However, further studies are required to establish the mechanism, underlying the augmentation of tissue antioxidants.

## Conclusions

The present study, for the first time, demonstrated that long term oral supplementation of palm olein oil caused augmentation of endogenous antioxidants of heart, which were subsequently protected from developing oxidative stress following ischemia-reperfusion.

## Methods

### Preparation of diet

Palm olein oil (Ruchi Gold, India) was obtained from the local market. Commercial rat diet (Ashirwad, India) containing protein: 24%, fat: 5%, fiber: 4%, carbohydrates: 55%, calcium: 0.6%, phosphorus: 0.3% w / w was supplemented with palm olein oil in two different doses [5 % v / w and 10 %v / w of diet]. The doses were selected from the previous studies [13,17]. Diet and water were provided *ad libitum*.

### Animals

The study was approved by Institute Animal Ethics Committee (245 / IAEC / 04) and all animal care and experimental protocols were in compliance with the NIH guidelines for the care and use of the Laboratory Animals (NIH Publication #85–23, 1985). Laboratory bred Wistar rats (150–200 gm) of either sex were maintained under standard laboratory conditions at temperature 25 ± 2°C, relative humidity 50 ± 15% and normal photo period (12 h dark / 12 h light) was used for the study.

### Chemicals

All chemicals were of analytical grade and chemicals required for sensitive biochemical assays were obtained from Sigma Chemicals (St. Louis, USA). Double distilled water (DDW) was used in all biochemical assays.

### Experimental protocol

After one week of acclimatization, rats were randomly divided into three groups, each group containing 16 rats. In control group (C), rats were fed with normal diet for 30 days. In groups (PO5 and PO10), rats were fed with palm olein oil supplemented commercial rat diet for 30 days in two different doses; 5% v / w and 10% v / w of diet. Changes in body weight, food and water intake patterns of rats in all the groups were noted throughout the experimental period. At the end of the 30 days, rats were fasted overnight and half the rats from each group were subjected either to protocol I or to protocol II as described below. Rats were heparinised (375 IU / 200 gms, i.p), and 0.5 h later rats were anesthetized with sodium pentobarbitone (60 mg / Kg, i.p) and euthanised.

### Protocol I

#### Basal level of biochemical and histopathological studies

Immediately after euthanization, the hearts were rapidly harvested, washed in ice cold saline, frozen in liquid nitrogen and stored at -80°C until processed for estimations of biochemical parameters. For histopathological studies, heart was stored in 10% buffered formalin (pH 7.2).

Group C: Normal diet fed rats (n = 8)

Group PO 5: 5% v / w palm olein oil supplemented diet fed rats (n = 8)

Group PO 10:10% v / w palm olein oil supplemented diet fed rats (n = 8)

### Protocol II

#### Production of *in vitro *ischemic reperfusion injury

Immediately after euthanization, hearts were rapidly harvested, washed in ice-cold saline, and perfused with the non-recirculating Langendorff's technique (Hufesco, Hungary), under constant pressure mode with modified Kreb Hensleit's buffer [18] containing [mM]: glucose 11.1; NaCl 118.5; NaHCO_3 _25; KCl 2.8; KH_2_PO_4 _1.2; CaCl_2 _1.2; MgSO_4 _0.6, with a pH of 7.4. The buffer solution equilibriated with 95% O2+ 5% CO2 was delivered to the aortic canula at 37°C and 65 mm Hg pressure. Following 10 min. of equilibration period, hearts were subjected to 20 min. of zero flow (global ischemia) and 40 min. of re-flow (reperfusion) [19,20].

Group C IR: Normal diet fed rats subjected to IR injury (n = 8)

Group PO5 IR: 5% v/w palm olein oil supplemented diet fed rats subjected to IR injury (n = 8)

Group PO10 IR: 10% v/w palm olein oil supplemented diet fed rats subjected to IR injury (n = 8)

At the end of each experiment, heart was frozen in liquid nitrogen and stored at -80°C until processed for estimations of biochemical parameters. For histopathological studies, heart was stored in 10% buffered formalin (pH 7.2).

### Biochemical parameters

#### Myocardial TBARS [21]

Hearts were homogenized in 10% trichloroacetic acid (TCA) at 4°C. 0.2 ml homogenate was pipetted into a test tube, followed by addition of 0.2 ml of 8.1 % sodium dodecyl sulphate (SDS), 1.5 ml of 30% acetic acid (pH-3.5), 1.5 ml of 0.8% thiobarbituric acid (TBA) and volume was made upto 4.0 ml with DDW. Test tubes were boiled at 95°C for 60 min. and then cooled. 1.0 ml of DDW and 5.0 ml of n-butanol: pyridine (15:1 v / v) mixture was added to the test tubes and centrifuge at the 4,000 × g for 10 min. The absorbance of developed colour in organic layer was measured at 532 nm.

Commercially available 1, 1, 3, 3-tetraethoxypropane (Sigma Chemicals) was used as a standard for MDA. Data is expressed as nmol / mg protein.

#### Myocardial CAT [22]

Hearts were homogenized at 4°C (1:10) in 50 mmol/l potassium phosphate buffer (pH- 7.4) and centrifuged at 3,000 × g for 10 min. Supernatant (50 μl) was added to a 3.0 ml cubette that contained 1.95 ml of 50 mM phosphate buffer (pH-7.0). Then1.0 ml of 30 mM hydrogen peroxide was added and changes in absorbance were measured for 30 sec. at 240 nm at an interval of 15 sec. Catalase activity is expressed as units / mg protein as compared to the standard.

#### Myocardial SOD [23]

Hearts were homogenized in 0.25 M tris sucrose buffer and centrifuged at 10,000 × g for 15 min at 4°C. The supernatant was fractionated by 50% ammonium sulphate and dialysed overnight. Aliquots of the supernatant (100 μl) was added to sodium pyrophosphate buffer (pH-8.3) followed by addition of 0.1 ml of 186 μM phenazine methosulphate, 0.3 ml of 300 mM nitroblue tetrazolium and 0.2 ml of 780 μM NADH. Reaction mixture was incubated for 90 sec. at 30°C and stopped the reaction by adding 1.0 ml of glacial acetic acid. 4.0 ml of n-butanol was then added and centrifuged at 3,000 × g for 10 min. The absorbance of organic layer was measured at 560 nm. SOD activity is expressed as units / mg protein as compared to the standard.

#### Myocardial GPx [24]

Hearts were homogenized at 4°C in 0.25 M phosphate buffer saline (pH-7.0). Homogenate was centrifuged at 15,000 × g for 60 min. at 4°C and supernatant were assayed for the GPx activity. GPx activity was in a 1.0 ml cubette containing 400 μl of 0.25 M potassium phosphate buffer (pH-7.0), 200 μl of sample, 100 μl of 10 mM GSH, 100 μl of 2.5 mM NADPH and 100 μl of glutathione reductase (6 U / ml). Hydrogen peroxide (100 μl of 12 mM) was then added and change in absorbance was measured at an interval of 1 min for 5 min at 366 nm. GPx activity is expressed as units / mg protein as compared to the standard.

Protein concentration was measured by Bradford method [25].

### Histopathological studies

Heart tissue was fixed in 10% buffered formalin, routinely processed and embedded in paraffin. Paraffin sections (3 μm) were cut on glass slides and stained with hematoxylin and eosin (H&E), periodic acid Schiff (PAS) reagent and examined under a light microscope (Nikon, Japan). Histopathological study was carried out by one of the authors (AKD), blinded to the groups.

### Statistical analysis

All values are expressed as mean ± SE. One way ANOVA followed by Bonferroni test was applied to test for significance of biochemical data of the different groups. Significance is set at p < 0.05.

## Abbreviations

IRI: ischemic-reperfusion injury; PO: palm olein oil; PO5: rats fed with 5% v/w palm olein oil supplemented diet; PO10: rats fed with 10% v/w palm olein oil supplemented diet; C: rats fed with normal diet; C IR: rats fed with normal diet subjected to ischemic-reperfusion injury; PO5 IR: rats fed with 5% v/w palm olein oil supplemented diet subjected to ischemic-reperfusion injury; PO10 IR: rats fed with 10% v/w palm olein oil supplemented diet subjected to ischemic-reperfusion injury; TBARS: thiobarbituric acid reactive substances; SOD: superoxide dismutase; CAT: catalase; GPX: glutathione peroxidase; IHD: ischemic heart disease; ROS: reactive oxygen species; PGI_2_: prostacyclin; TXA_2_: thromboxane A_2 _; SE: standard error; ANOVA: analysis of variance; MDA: malondialdehyde; TCA: tricarboxylic acid; SDS: sodium dodecyl sulphate; DDW: double distilled water; NADH: nicotinamide adenine dinucleotide reduced; TBA: thiobarbituric acid; NADPH: nicotinamide adenine dinucleotide phosphate reduced; GSH: reduced glutathione; PAS; periodic acid Schiff reagent; H&E: hematoxylin and eosin.

## Authors' contributions

DN carried out the animal experimentation, biochemical estimation and statistical analysis of results. SS and MT participated in the design of the study and statistical analysis. AKD carried out the light microscopic study. SKM conceived the study, participated in its design and coordination and drafted the manuscript. All authors read and approved the final manuscript.
